# Integrative Evaluation of Bead Morphology in Plasma Transferred Arc Cladding Through Orthogonal Arrays and Morphology Index Analysis

**DOI:** 10.3390/ma18225155

**Published:** 2025-11-13

**Authors:** Lihe Jiang, Jinwei Long, Yanhong Wei, Qian Jiang, Fangxuan Wang

**Affiliations:** 1 College of Materials Science and Technology, Nanjing University of Aeronautics and Astronautics, 29 Jiangjun Rd., Nanjing 211106, China; jung2306502@nuaa.edu.cn (L.J.); jinweilong@nuaa.edu.cn (J.L.); 2 Cosco Shipping Marine Equipment & Spares (Nanjing) Co., Ltd., 18 Tangwei Rd., Nanjing 211121, Chinawang.fangxuan@coscoshipping.com (F.W.); 3 Jiangsu Engineering Technology Research Center for Advanced Manufacturing of Ship Power System Components, Nanjing 211121, China

**Keywords:** plasma transferred arc (PTA), bead morphology, orthogonal design, morphology index, process optimization, additive manufacturing

## Abstract

Plasma Transferred Arc (PTA) cladding is a versatile hardfacing technique that produces dense, metallurgically bonded overlays with excellent wear and corrosion resistance. However, optimizing bead shape is challenging due to complex multi-parameter interactions, an issue not fully addressed in existing studies. The bead morphology, defined by height, width, and penetration depth, remains highly sensitive to process parameters, directly affecting dilution and overall coating quality. In this work, single-pass powder PTA cladding was systematically studied using an orthogonal experimental design to assess the effects of arc current, powder feed rate, welding speed, oscillation width, and oscillation speed. A morphology index was proposed to integrate geometric attributes into a single metric for quality evaluation. Regression analysis and finite element simulations based on a Goldak double-ellipsoid heat source revealed that arc current is the dominant factor, where low-to-moderate values (100–115 A) promote wide–shallow pools and higher morphology index values, while higher currents induce excessive penetration and reduced stability. Multi-parameter coupling further indicated that optimal bead morphology is achieved under low-to-moderate current, a high welding speed, relatively high powder feed rate, wide oscillation width, and moderate oscillation speed. A representative optimal condition (100 A, 105 mm·min^−1^, 35 g·min^−1^, 10 mm, 2600 mm·min^−1^) ensured minimal dilution and stable deposition. This integrative framework of orthogonal design, morphology index evaluation, and thermo-fluid simulation provides practical guidelines for parameter optimization and represents a novel combined approach for PTA bead optimization.

## 1. Introduction

**Figure 1:**
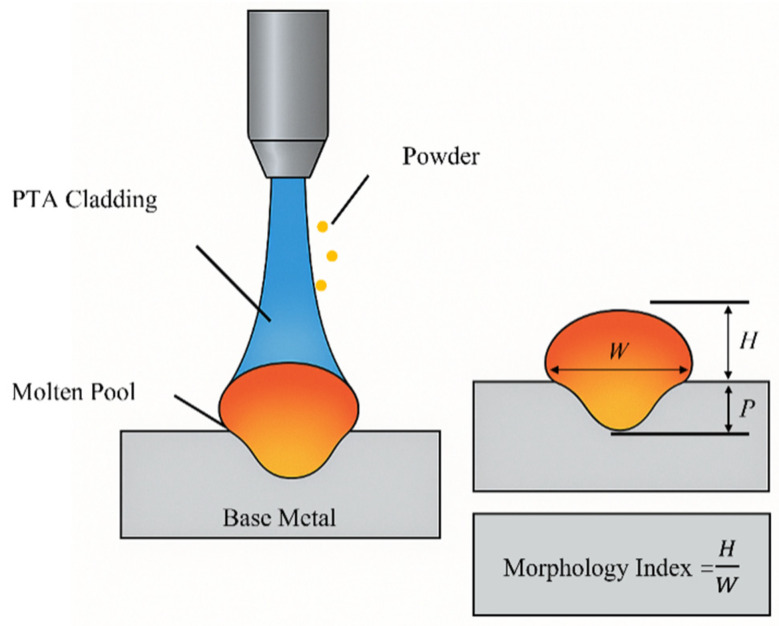
Schematic illustration of the PTA cladding process and key geometric parameters of the weld bead: height (*H*) refers to the buildup thickness of the clad above the substrate surface; width (*W*) represents the lateral span of the deposited bead; and penetration depth (*P*) denotes the depth to which the molten material has fused into the substrate. These three parameters are used for morphology index evaluation.

The plasma transferred arc (PTA) hardfacing process has emerged as a versatile method for depositing metallurgically bonded overlays that enhance wear, corrosion and thermal resistance on critical components. Compared with traditional gas-shielded welding techniques, PTA offers higher energy density, stable powder delivery and precise heat input control, enabling thin, uniform deposits with minimal dilution and good surface finish [1]. These attributes underpin its widespread adoption in industries such as valve manufacture, hydraulic machinery, mining and power generation. Because the quality of a PTA overlay is largely defined by the weld bead geometry, characterized by height, width, and penetration depth, accurate control of these geometric parameters is essential. The bead shape influences dilution, microstructure and mechanical properties; thus, process parameters must be established and categorized to enable automation and to ensure uniform, defect-free claddings [2]. To this end, a systematic approach that integrates multiple geometric responses into a single morphology index is desirable for quantitative assessment and optimization of bead quality, as illustrated in Figure 1.

Extensive international research has focused on optimizing plasma transferred arc (PTA) process parameters. Dasgupta et al. [3] employed a central composite design and regression analysis to investigate the influence of variables such as welding current, travel speed, and powder feed rate on Colmonoy overlays, with the objective of reducing penetration and enhancing bead geometry. Prabhakaran et al. [4] utilized Taguchi design of experiments and identified dilution as a critical factor, demonstrating that arc current and stand-off distance significantly affect bead quality. More recently, Zanzi et al. [5] studied Inconel 625 cladding and found that stand-off distance and carrier gas flow were the most influential parameters for deposition efficiency, achieving a dilution rate as low as 9% under optimal conditions. These studies collectively validate the effectiveness of statistical optimization techniques in enhancing PTA performance. However, most focus on individual response variables and do not provide an integrated evaluation of overall bead morphology.

In plasma-transferred-arc (PTA) hardfacing, the alloying composition of the filler powder critically governs the clad microstructure and properties. Nickel-based Ni–Cr–B–Si alloys (for example, Colmonoy 56) produce a Ni-rich matrix containing dense chromium boride and carbide precipitates. The resulting hard intermetallic network greatly increases the hardness and abrasion resistance of the deposit, while the nickel matrix affords good corrosion resistance. By contrast, high-carbon Fe–Cr–V–C powders solidify to form primary vanadium carbide (VC) and chromium carbide (M_7_C_3_) phases throughout the microstructure. This Fe–Cr–V–C hardfacing is tailored for extreme wear service: the in situ VC/Cr-carbide network produces very high hardness and toughness. However, because the matrix is iron-based, its corrosion resistance is generally lower than that of a Ni–Cr–B–Si coating. Thus, powder selection dictates a key trade-off between wear resistance and corrosion resistance. Ni–Cr–B–Si claddings tend to deliver both high wear resistance and good corrosion protection, whereas Fe–Cr–V–C claddings maximize abrasive wear resistance (often at lower cost) at the expense of corrosion resistance. Careful choice of the PTA hardfacing powder is therefore essential to balance the overlay’s wear and corrosion performance.

In China, PTA research has focused on improving material performance and developing adaptive control systems. Peiran Shi et al. [6] at Wuhan University used PTA to deposit Ni–Cr–Mo coatings with varying Mo contents. Their microstructural analysis showed that the addition of 5% Mo led to granular austenite precipitates and significantly enhanced both wear and corrosion resistance. Xin Zhang et al. [7] at Central South University prepared Fe–Cr–V–C composite coatings via PTA powder surfacing and systematically examined how powder composition and process parameters affect hardness and microstructure, providing guidance for the design of iron-based composite overlays. Its high hardness arises from a Ni-rich matrix embedded with Cr-rich boride and carbide phases, providing strong wear and abrasion resistance. Nan Li et al. [8] proposed a double-electrode micro-PTA additive manufacturing system with a self-adaptive control strategy. By dynamically adjusting the wire feed rate and torch stand-off distance, they stabilized the process and improved layer appearance. While these domestic studies advance coating performance and process control, they primarily focus on microstructural characterization or system design, and do not comprehensively evaluate how individual or coupled process variables influence single-pass bead geometry.

Despite these advances, two important gaps remain. First, most investigations target multipass deposition or micro-PTA variants; the systematic studies of single-pass PTA bead morphology, where parameter sensitivity is more pronounced, are scarce. Second, existing optimization strategies typically consider individual responses (e.g., dilution or reinforcement) but lack a unified morphology index that captures the coupled effects of height, width and penetration. Without such an index, it is difficult to quantify the trade-offs between geometric attributes and to identify parameter windows that simultaneously minimize dilution and maximize surface integrity [9,10]. There is therefore a need for a framework that combines orthogonal experimental design, morphology index evaluation and numerical simulation to elucidate the thermofluid mechanisms governing molten pool behavior and to derive practical guidelines for parameter selection [11,12,13].

The present study addresses these gaps by systematically analyzing the influence of arc current, powder feed rate, welding speed, oscillation width and oscillation speed on bead morphology in powder PTA cladding. An integrated morphology index is proposed to quantify geometric quality by combining bead height, width and penetration depth, providing a holistic measure of deposition performance. Orthogonal arrays are employed to efficiently explore the multidimensional parameter space, and a regression-based evaluation model is developed to identify dominant factors and interactions. In parallel, a finite-element thermal–fluid model based on the Goldak double-ellipsoid heat source is used to simulate molten pool evolution and to validate experimental observations. By integrating experiments and simulations, the study reveals that arc current is the most influential parameter, and that optimal morphology is achieved at low-to-moderate current combined with high welding speed, relatively high powder feed rate, wide oscillation width and moderate oscillation speed. The resulting guidelines and insights into molten pool dynamics provide a foundation for adaptive process control and advance the state of knowledge on single-pass PTA cladding. To systematically evaluate the effects of key process parameters on single-pass bead geometry, to develop a unified morphology index combining height, width, and penetration, to model the thermal-fluid behavior of the PTA pool, and to derive practical parameter guidelines.

## 2. Materials and Methods

### 2.1. Materials and PTA Cladding Setup

In this study, the arc current was systematically varied across experiments, while the arc voltage remained approximately constant at 30–32 V, as measured at the torch terminals. The effective power input (Q) was estimated using the expression Q = V·I·η, where η represents the thermal efficiency of the arc process, assumed to be ~0.7 based on prior PTA studies. Since the nozzle geometry constrained the arc cross-sectional area, current served as the primary control variable influencing arc energy density and heat input.

Alloy 56 (Colmonoy 56) is a nickel-based hardfacing powder extensively employed in plasma transferred arc (PTA) surfacing owing to its ability to produce dense and metallurgically bonded overlays with reliable wear and corrosion resistance. The alloy is specifically formulated to promote the precipitation of chromium carbides, which are primarily responsible for abrasion and corrosion resistance. In addition, controlled additions of boron and silicon improve weldability and contribute to the stabilization of the molten pool during deposition. The powder is manufactured by gas atomization, resulting in spherical particles with low inclusion content and favorable flowability, which facilitates uniform feeding and smooth spreading during the cladding process. The chemical composition of Alloy 56 is presented in Table 1. The chemical composition of Alloy 56 is presented in Table 1. The composition was measured using X-ray fluorescence (XRF) spectroscopy (AxiosMAX, PANalytical B.V., Almelo, The Netherlands), which provides accurate elemental quantification for alloy powders. When deposited by PTA, this material typically produces hard, crack-free coatings with low dilution. Single-pass overlays generally exhibit hardness values in the range of 46–51 HRC, whereas double-pass layers reach 53–58 HRC. These characteristics render Alloy 56 a suitable candidate for the protection and refurbishment of mechanical components operating in severe service environments, where long-term wear and corrosion resistance are required.

45Cr9Si3 electroslag remelted steel was selected as the substrate material. This martensitic valve steel is widely used in gas valve applications because it combines high strength with excellent resistance to heat, wear, and oxidation. Its composition, summarized in Table 2, is designed to ensure good high-temperature stability and corrosion resistance. The elemental composition of the 45Cr9Si3 electroslag remelted steel substrate was determined by optical emission spectroscopy (OES) using a SpectroMAXx (SPECTRO Analytical Instruments GmbH, Kleve, Germany). When properly heat-treated, the alloy exhibits a yield strength of approximately 790 MPa and a Brinell hardness of about 433. The Brinell hardness scale (HB) was used for the substrate due to its thick section and relatively moderate hardness, making it suitable for bulk steel. In contrast, the Rockwell C scale (HRC) was employed for the much harder surface cladding, as it is more sensitive and accurate in high-hardness measurements. These attributes make 45Cr9Si3 electroslag remelted steel an ideal foundation for hardfacing: its high-temperature strength and thermal compatibility help to minimize thermal stresses during plasma cladding, while the resulting composite structure provides a wear- and corrosion-resistant surface layer without compromising the substrate’s mechanical integrity.

**Figure 2:**
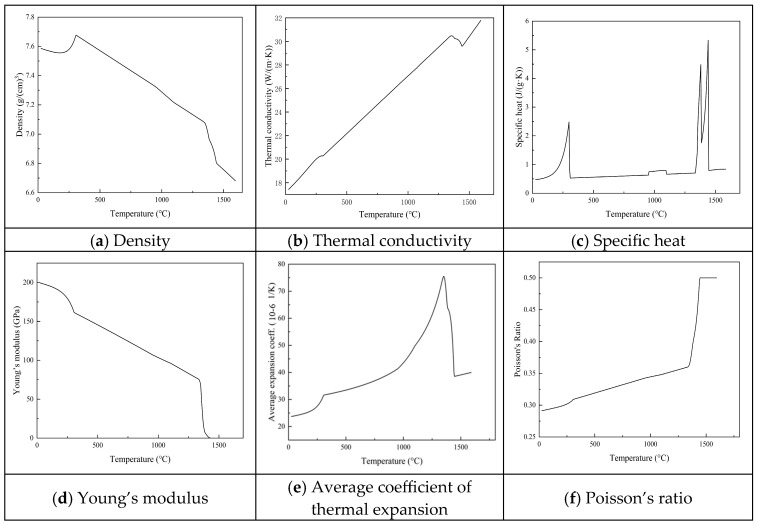
Thermophysical properties of 45Cr9Si3 steel used in the finite element simulation: (**a**) density (g/m^3^); (**b**) thermal conductivity (W/m·K); (**c**) specific heat capacity (J/kg·K); (**d**) Young’s modulus (GPa); (**e**) coefficient of thermal expansion (10^−6^/K); and (**f**) Poisson’s ratio.

The temperature-dependent thermophysical properties, including density, thermal conductivity, specific heat capacity, Young’s modulus, the average coefficient of thermal expansion, and Poisson’s ratio, were calculated using JMatPro v12.1 and employed as input parameters for modeling the molten pool morphology and thermo-mechanical behavior during the PTA cladding process. Figure 2 illustrates the variation in these properties with temperature for the substrate material 45Cr9Si3 electroslag remelted, where panels (a)–(f) correspond to density, thermal conductivity, specific heat capacity, Young’s modulus, average thermal expansion coefficient, and Poisson’s ratio, respectively.

**Figure 3:**
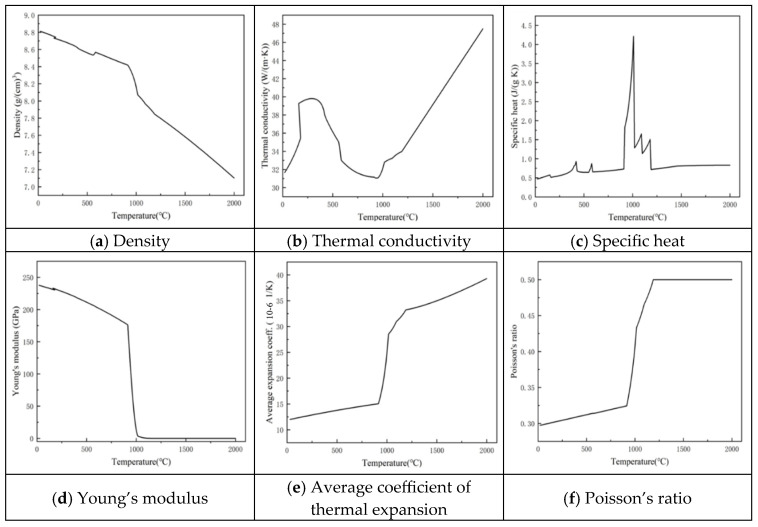
Thermophysical properties of Colmonoy 56 alloy powder: (**a**) density (g/m^3^); (**b**) thermal conductivity (W/m·K); (**c**) specific heat capacity (J/kg·K); (**d**) Young’s modulus (GPa); (**e**) coefficient of thermal expansion (10^−6^/K); and (**f**) Poisson’s ratio.

Similarly, Figure 3 presents the corresponding temperature-dependent trends for the cladding powder Alloy 56 (Colmonoy 56), with panels (a)–(f) arranged in the same order for direct comparison.

The plasma-transferred arc (PTA) cladding experiments were carried out using a fully automated surfacing system, which integrated a power supply, a numerically controlled six-axis robotic arm for precise torch manipulation, a water-cooled plasma torch, a powder feeding unit, a motorized workpiece table, temperature monitoring devices, and gas delivery subsystems. The PTA torch was equipped with a non-consumable tungsten cathode and a water-cooled copper anode containing a constricting orifice, which served to accelerate and focus the plasma jet. A shielding nozzle was positioned concentrically around the arc zone to protect the molten pool from atmospheric contamination.

To initiate and stabilize the arc, separate power sources were employed for the main arc and the pilot arc. The pilot arc, sustained between the cathode and anode, ensured consistent and reliable ignition of the transferred arc between the cathode and the workpiece surface. During operation, three distinct gas flows were utilized: the plasma gas, composed of argon or an argon–hydrogen mixture, was ionized and expelled through the anode orifice to generate the high-temperature plasma jet; the shielding gas was introduced to maintain an inert atmosphere around the arc and molten pool, thereby preventing oxidation; and the carrier gas was used to convey the alloy powder from the feeder into the plasma stream for deposition.

**Figure 4:**
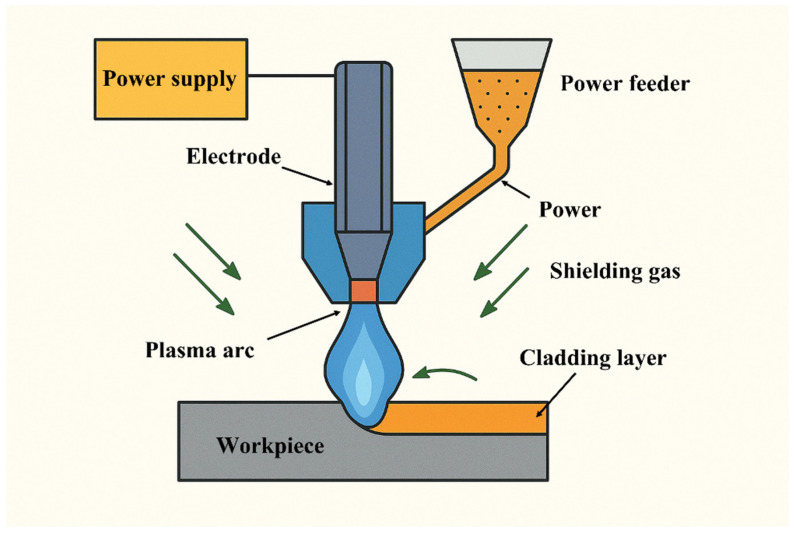
Photograph of the experimental PTA cladding setup: (1) plasma torch; (2) powder feed nozzle and feeder; (3) six-axis robotic manipulator arm; (4) moving workpiece table.

A gas mixture of argon containing 2–6 vol.% hydrogen was selected to create a mildly reducing atmosphere, which enhances the thermal efficiency of the arc and improves the metallurgical bonding between the deposited material and the substrate. A photograph of the cladding apparatus is shown in Figure 4, highlighting the key components: (1) the plasma torch, (2) the powder feeder and nozzle, (3) the robotic manipulator arm, and (4) the moving workpiece table.

Prior to deposition, the 45Cr9Si3 electroslag remelted steel coupons (100 mm × 50 mm × 10 mm) were precision-machined to the desired dimensions, followed by surface preparation involving mechanical grinding to eliminate oxides and machining marks. Subsequently, the samples were degreased in acetone and preheated to 150 °C for 30 min, which moderately reduces thermal gradients and helps reduce residual stresses

The selection of process parameters for the pulsed plasma transferred arc (PTA) surfacing experiments was guided by parameter windows reported in the existing literature. Published studies on similar PTA-based overlay systems have typically employed upper torch currents ranging from 110 A to 190 A, traverse speeds between 0.4 and 0.8 mm·s^−1^, transferred arc currents in the range of 100–180 A, powder feed rates from 5 to 13 g·min^−1^, and welding speeds of 70–190 mm·min^−1^. Drawing from these benchmarks, appropriate values for arc current, travel speed, powder feed rate, and shielding/powder carrier gas flow rates were selected to ensure process stability and to achieve a desirable bead geometry suitable for the Alloy 56/45Cr9Si3 electroslag remelted material system. The specific parameters employed in this study are summarized in Table 3.

The selected process parameters ensured the formation of a stable and well-confined molten pool, with controlled dilution between the clad layer and the substrate. All parameter values fell within the established operating window for plasma transferred arc (PTA) surfacing, as reported in prior literature, thereby promoting process stability and metallurgical integrity. Upon completion of the cladding process, the specimens were allowed to cool naturally under a flowing argon atmosphere to suppress oxidation and minimize the development of residual stresses prior to subsequent mechanical characterization.

### 2.2. Process Parameters and Design of Experiments

To evaluate the influence of process variables on the quality and consistency of PTA cladding using Alloy 56 on a 45Cr9Si3 electroslag remelted substrate, a structured design of experiments (DOE) approach was adopted. Considering equipment limitations and the need for stable arc behavior, a modified orthogonal experimental matrix was constructed. This design allowed the efficient investigation of key process factors while avoiding redundant or impractical parameter combinations. The parameter levels were arranged in an orthogonal array using the L_9_(3^4^) design matrix. The parameter ranges and levels were selected based on prior literature values and preliminary trial runs to ensure feasibility and adequate coverage of the expected bead morphology variations.

The five key process parameters considered were: arc current (A), powder feed rate (g/min), welding speed (mm/min), torch oscillation speed (mm/min), and oscillation width (mm). Among these, the arc current was selected as the primary variable and varied across five levels from 100 A to 160 A. Other parameters were held constant across all experimental runs to isolate the effects of current variation: the powder feed rate was fixed at 25 g/min to maintain a consistent material input, welding speed was set at 88 mm/min to control heat input per unit length, the torch oscillation speed was fixed at 2600 mm/min to ensure uniform bead spreading, and the oscillation width was maintained at 8.0 mm for consistent overlap.

The detailed combinations of parameters used in this study are presented in Table 4, which outlines the experimental conditions for each run. This design strategy ensured that the trials remained within the established PTA processing window, while providing meaningful insights into the influence of current variation under otherwise constant deposition conditions.

### 2.3. Construction of Evaluation Metrics for PTAW Bead Geometry

In the context of Plasma Transferred Arc Welding (PTAW), achieving a high-quality weld bead requires precise control over several process parameters, each of which influences the geometry of the deposited layer. The weld bead’s geometric morphology—typically characterized by height, width, and penetration depth—plays a critical role in determining the mechanical performance, dilution, and surface integrity of the cladding layer. Therefore, developing a robust evaluation index that correlates process variables with geometric outcomes is essential for process optimization.

#### 2.3.1. Influence of Process Parameters on Bead Geometry in PTAW

Numerous studies employing ANOVA, regression modeling, and gray relational analysis have consistently demonstrated the critical influence of key process parameters on weld bead geometry in Plasma Transferred Arc Welding (PTAW). Among these, welding speed (*v*) has been identified as the most dominant factor. An increase in torch traverse speed typically reduces the heat input per unit length, resulting in decreased bead height and width, while simultaneously increasing the dilution ratio due to deeper penetration and thinner cladding layers [14,15]. The powder feed rate (g/min) also plays a significant role, particularly in determining bead height. A higher feed rate enhances material deposition and energy absorption, thereby increasing bead height and reducing dilution; however, excessively high feed rates can compromise the width-to-height ratio and introduce instability. Welding current (*I*) primarily affects the penetration depth, with higher currents generating greater arc energy and thus elevating the melt pool temperature and depth, though having relatively minor effects on bead width and height. Additionally, oscillation parameters—including oscillation speed and width—affect the thermal field distribution and wetting behavior. Increased oscillation width generally expands the bead width while reducing both penetration depth and bead height, leading to improved surface flatness but potentially increasing bead asymmetry if not carefully controlled.

#### 2.3.2. Regression-Based Bead Morphology Evaluation

To quantitatively assess the geometric quality of PTA weld beads, a morphology index (*M*) is defined based on the three key geometric attributes:

(1) $$M=\frac{H\cdot W}{P}$$

where *H* is bead height, *W* is bead width, and *P* is penetration depth. A higher value of *M* implies a desirable geometry characterized by high buildup and wide coverage with minimal dilution.

Based on the experimental data obtained from PTAW (Plasma Transferred Arc Welding) processes, second-order regression models have been developed to characterize the relationships between bead geometry and five key process parameters: welding current (*A*), powder feed rate (*F*), welding speed (*S*), oscillation width (*O*), and oscillation speed (*T*). The models are given by:

(2) p=0.9506+0.252A−0.038O−0.107S−0.134F+⋯H=3.514−0.003A−0.179O−0.074S+0.020T+0.048F+⋯W=18.96+0.856A+1.11O−0.346S−0.220T+0.093F+⋯

By substituting Equation (2) into Equation (1), a process-parameter-based morphology index is obtained:

(3) $$M\left(A,F,S,v_{osc},w_{osc}\right)=\frac{H(A,w_{osc},S,v_{osc},F)\cdot W(A,w_{osc},S,v_{osc},F)}{P(A,w_{osc},S,v_{osc},F)}$$

This model enables predictive evaluation of bead geometry under specific process conditions and can be used to determine optimal parameter settings for minimal dilution and maximal deposition efficiency in PTA cladding operations.

#### 2.3.3. Dimensionless Evaluation Model for PTA Process Optimization

In cases where regression data is limited or unavailable, a normalized, dimensionless morphology index (*M_norm_*) can serve as an effective alternative. This model uses relative parameter weighting to estimate the proximity of current settings to optimal conditions:

(4) $$M_{norm}={\left(\frac{I}{I_{ref}}\right)}^{\gamma_{1}}{\left(\frac{\dot{m}}{{\dot{m}}_{ref}}\right)}^{\gamma_{2}}{\left(\frac{v_{ref}}{v}\right)}^{\gamma_{3}}{\left(\frac{v_{osc},ref}{v_{osc}}\right)}^{\gamma_{4}}{\left(\frac{w_{osc},ref}{w_{osc}}\right)}^{\gamma_{5}}$$

The variables *I* and *I_ref_* represent the welding current and its reference value, respectively. The symbol $\dot{m}$ denotes the wire feed rate, with ${\dot{m}}_{ref}$ as its desired value. The parameter *v* indicates the welding speed, while *v_osc_* and *w_osc_* represent the oscillation speed and amplitude, respectively. The weight coefficients *γ*_i_ reflect the relative importance of each parameter. According to the sensitivity analyses reported in the literature, the priority ranking of parameter importance is *γ*_3_ > *γ*_2_ > *γ*_1_ > *γ*_4_ > *γ*_5_.

Based on sensitivity rankings observed in PTAW literature, the recommended weights are:

$$\gamma_{3}=2,\;\gamma_{2}=1.5,\;\gamma_{1}=1,\;\gamma_{4}=0.5,\;\gamma_{5}=0.5$$

Higher values of *M_norm_* suggest that the selected parameter set is closer to the reference condition associated with optimal bead morphology. This model is particularly useful for the preliminary screening or real-time feedback control in automated PTA systems.

This study introduces two complementary models for evaluating the geometric morphology of weld beads in PTAW: a regression-based index (Equation (3)) that enables accurate morphology prediction and parameter optimization based on empirical data, and a dimensionless normalized index (Equation (4)), which facilitates rapid assessment and real-time control in scenarios lacking experimental models. These models are broadly applicable across various PTA cladding processes and hold potential for integration into closed-loop control systems or digital twins. Future developments may involve refining the weighting coefficients and regression structures through expanded datasets or machine learning-driven modeling strategies.

## 3. Numerical Simulation Framework

### 3.1. Governing Equations and Material Constitutive Models

Numerical simulation of plasma transferred arc (PTA) cladding treats the molten pool as an incompressible Newtonian fluid whose behavior is governed by the conservation of mass, momentum and energy. In the general form adopted by this study, the mass (continuity) equation ensures that the divergence of velocity is zero in the liquid region. The momentum equations correspond to the Navier–Stokes equations; the transient inertial term, convection term, and viscous term are balanced by pressure gradients and source terms. These source terms include buoyancy forces (implemented by the Boussinesq approximation), shear stress due to surface-tension gradients (Marangoni force) and recoil pressure from metal vapor; such forces are recognized as the main drivers of flow in the molten pool. To model melting and solidification, the enthalpy–porosity method is used: a liquid fraction *f_l_* derived from local temperature defines an additional Darcy-type damping term in the momentum equations; this term gradually suppresses velocity as the material solidifies, allowing the mushy zone to be modeled without explicitly tracking the solid–liquid interface.

The energy equation is formulated in terms of temperature and accounts for conduction, convection and latent heat. It can be written as

(5) $$\rho c_{p}\left(\frac{\partial T}{\partial t}+v\cdot \nabla T\right)=\nabla \cdot \left(k\nabla T\right)+Q-\rho L\frac{\partial f_{l}}{\partial t}$$

where *ρ* is density, *c_p_* is specific heat, *k* is thermal conductivity, *Q* represents volumetric heat sources (including the external heat source and viscous dissipation) and *L* is latent heat. The latent heat term couples the energy equation to the liquid-fraction evolution and enables the simulation to capture melting and solidification. Surface boundary conditions combine convective and radiative heat losses: the net heat flux at exposed surfaces is *q_loss_* = *h_c_* (*T* − *T_R_*) + *σ* (*T*^4^ − *T_R_*^4^), where *h_c_* is the convection coefficient, *T_R_* is the ambient temperature and *σ* is the Stefan-Boltzmann constant; evaporative heat loss is also considered when the surface temperature exceeds the evaporation temperature. On the pool surface, a stress boundary condition accounts for the temperature dependence of surface tension: the tangential shear stress is *τ_s_* = *∂σ/∂T*·∇_‖_T, and the pressure jump includes recoil pressure from metal vapor.

Material properties are modelled as temperature-dependent. Density *ρ*(*T*), thermal conductivity *k*(*T*), specific heat *c_p_*(*T*) and dynamic viscosity *μ*(*T*) for the clad material and substrate are defined using experimentally reported curves. For example, in one study of molybdenum alloys fabricated by selective laser melting, the thermal conductivity of the powder bed is much lower than that of the solid metal and is simplified according to an empirical formula that considers porosity and radiation amongst particles. The viscosity and surface tension are also defined as functions of temperature, with a negative temperature coefficient of surface tension—this enables accurate representation of Marangoni convection. The damping constant used in the enthalpy–porosity method (often around 10^4^–10^6^) is chosen to ensure a sharp transition between liquid and solid phases.

### 3.2. Heat Source Modeling in PTA Cladding

To accurately model the highly concentrated energy input characteristic of the Plasma Transferred Arc (PTA) process, a volumetric heat source based on the Goldak double ellipsoid model is employed. This semi-empirical model effectively captures the three-dimensional distribution of heat in the weld pool and is particularly suitable for arc-based processes involving asymmetrical thermal fields. The model divides the heat source into front and rear ellipsoidal regions, each described by its own semi-axes: *a*, *b*, *c*_1_ (front), and *c*_2_ (rear). The total heat input *Q* is distributed between these regions via two parameters, *f_f_* and *f_r_*, such that *f_f_* + *f_r_* = 2, ensuring energy conservation.

The power density distribution in the front quadrant of the moving coordinate system

*ξ*, which translates along the scanning direction, is given by:

(6) $$q_{f}(x,y,z)=\frac{6\sqrt{3}f_{f}Q}{\pi abc_{1}}exp(-3\frac{\xi^{2}}{a^{2}}\;-\;3\frac{y^{2}}{b^{2}}\;-\;3\frac{z^{2}}{c_{1}^{2}})$$

Similarly, in the rear quadrant, the power density is defined as:

(7) $$q_{r}\left(x,y,z\right)=\frac{6\sqrt{3}f_{r}Q}{\pi abc_{2}}exp(-3\frac{\xi^{2}}{a^{2}}\;-\;3\frac{y^{2}}{b^{2}}\;-\;3\frac{z^{2}}{c_{1}^{2}})$$

**Figure 5:**
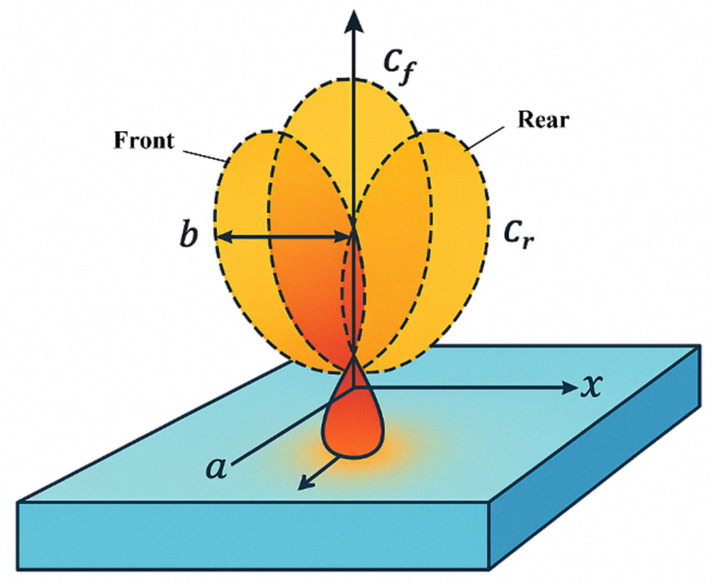
Schematic of the Goldak double-ellipsoid heat source model.

Here, *Q* = *η V I* represents the total heat input from the arc, where *V* is the arc voltage, *I* is the welding current, and *η* is the thermal efficiency of the heat source, typically ranging from 0.6 to 0.8 for PTA processes. The model parameters *a* and *b* define the width and depth of the heat source, respectively, while *c*_1_ and *c*_2_ allow asymmetry in the thermal field to be accurately captured [16]. The schematic of the double ellipsoidal heat source model is illustrated in Figure 5.

During simulation, the volumetric heat flux *q*(*x*,*y*,*z*) acts as a source term in the energy conservation equation, and is dynamically updated at each time step based on the current position of the heat source along the predefined scanning path. Calibration of the model is typically performed using experimental weld bead profiles, thermal measurements, or cross-sectional metallographic analysis to ensure predictive accuracy.

In contrast to laser cladding, the PTA process introduces additional complexities due to strong interactions between the arc and the powder stream, which may result in energy losses. Although effects such as shielding gas convection and arc plasma shear stress can be neglected or incorporated via user-defined subroutines, they are often omitted in baseline simulations. For instance, in the finite element study conducted by Senthiil and Shirrushti, forced convection effects due to shielding gas were not considered. However, at higher current levels, electromagnetic phenomena such as Lorentz force-driven stirring may become non-negligible and can be incorporated into the momentum equations to capture convective flows within the melt pool [17,18].

Typical heat input values employed in PTA simulations range from 1.0 to 1.5 kW, with double ellipsoidal parameters chosen to reflect the physical characteristics of the arc. Based on literature and calibration studies, representative parameter values are often within the following ranges: a ≈ 1.5–3.0 mm; b ≈ 2.0–3.0 mm; *c*_1_ and *c*_2_ selected based on experimental penetration depth and width.

Such detailed modeling facilitates the prediction of thermal fields, melt pool geometry, and resultant microstructure in PTA cladding processes, serving as a critical foundation for process optimization and quality control.

### 3.3. Melt Pool Evolution and Morphology Description

The time-dependent evolution of the molten pool is governed by the coupled phenomena of heat transfer and fluid flow. At the initial stage of heating, the arc energy rapidly elevates the local temperature above the melting point, initiating the formation of a small molten region. As the heat source traverses the substrate, this region expands and elongates [19]. Heat conduction into the surrounding solid material and convective transport within the liquid phase redistribute thermal energy, thereby shaping the evolving geometry of the melt pool. Fluid motion is primarily driven by Marangoni convection, induced by surface tension gradients, buoyancy forces resulting from temperature-dependent density variations, and recoil pressure generated by metal vaporization. These mechanisms collectively give rise to complex vortical flow structures that enhance fluid mixing within the melt pool. Such dynamic interactions may either stabilize or destabilize the molten surface, consequently affecting the final bead morphology [20,21].

**Figure 6:**
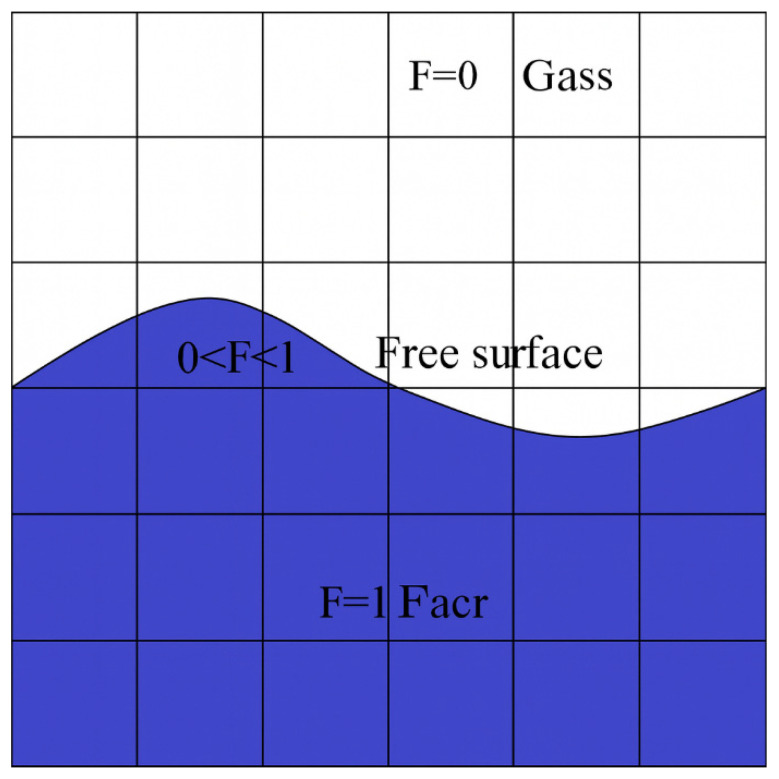
Schematic diagram of fluid volume function method.

To accurately capture the transient behavior of the melt pool’s free surface, numerical simulations employ interface tracking techniques such as the Volume of Fluid (VOF) method or the height function approach. In this framework, a scalar field representing the liquid volume fraction (denoted as *F*) is used to distinguish phases, where *F* = 1 corresponds to fully molten regions and *F* = 0 denotes solid material. A schematic illustration of the fluid volume function method is provided in Figure 6 to aid in conceptual understanding of the interface representation [22].

The molten pool plays a critical role in linking process parameters to final clad properties. Its shape and flow behavior are strongly influenced by thermal and fluid dynamic conditions. Key geometric descriptors such as pool width, depth, aspect ratio, and wetting angle evolve during processing. Higher current inputs lead to deeper and wider pools due to increased energy input, whereas faster scanning speeds or larger oscillation widths reduce interaction time, resulting in shallower and narrower melt pools. Powder feed rate also affects pool size, as excessive feed reduces the available energy for melting.

**Figure 7:**
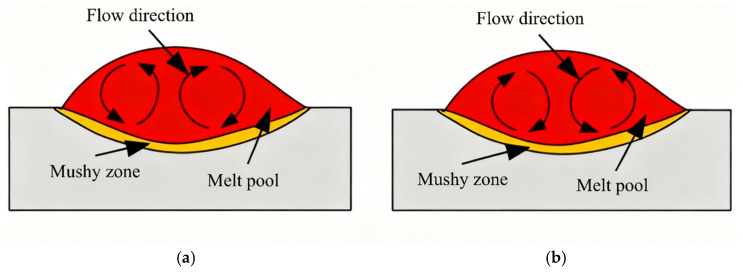
The influence of Marangoni coefficient on the flow direction of molten pool: (**a**) dγ/dT < 0; (**b**) dγ/dT > 0.

Flow within the molten pool is primarily governed by Marangoni convection, driven by surface tension gradients. The direction of this flow depends on the sign of the Marangoni coefficient. As shown in Figure 7, a negative coefficient (a) causes outward surface flow, while a positive coefficient (b) leads to inward circulation. These variations influence the distribution of heat and molten material, thereby affecting the stability and geometry of the melt pool [23,24].

The evolution of the molten pool directly impacts the solidification process and final morphology of the cladding layer. Cooling rates and thermal gradients in the mushy zone affect dendritic growth and grain structure. Accurate simulation and analysis of melt pool behavior allow for the prediction of solidification modes, such as the columnar-to-equiaxed transition (CET), and help identify potential defects like porosity, underfill, and segregation. Therefore, understanding melt pool dynamics is essential for optimizing process parameters and achieving desirable clad layer quality in PTA cladding.

## 4. Thermo-Mechanical Modeling and Molten Pool Morphology in Plasma Transferred Arc (PTA) Cladding

### 4.1. Development of a Thermo-Mechanical FEM Model for Single-Track PTA Cladding

In order to accurately simulate the thermal behavior and molten pool geometry during single-track Plasma Transferred Arc (PTA) cladding, a three-dimensional transient finite element model was developed. This model is designed to capture the heat transfer characteristics and dynamic formation of the molten pool under localized, highly concentrated plasma arc energy input.

**Figure 8:**
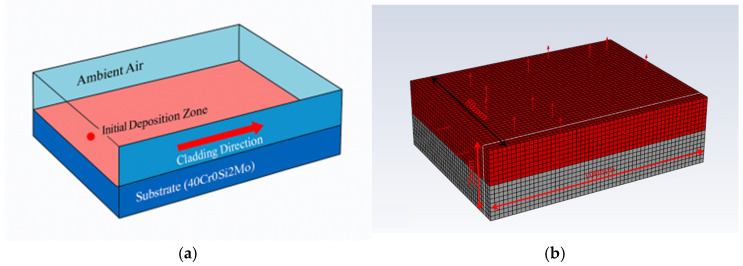
Schematic illustration of the geometric model and mesh discretization for laser cladding. (**a**) geometric model; (**b**) Mesh model.

As illustrated in Figure 8, the computational domain consists of a rectangular substrate made of 40CrSi2Mo steel, onto which a single clad track is deposited. The geometric model, shown in Figure 8a, includes the substrate, the initial deposition zone, and the surrounding ambient air. The clad layer advances progressively along the cladding direction, beginning from the initial deposition point, as indicated by the arrow.

The heat source is modeled using the Goldak Double-Ellipsoid Heat Source Model, a volumetric heat source formulation that more accurately captures the three-dimensional heat distribution typically observed in PTA processes. This model divides the heat source into front and rear ellipsoidal zones with independently defined geometrical parameters and heat intensity distribution, enabling better replication of the asymmetry in thermal gradients during forward motion of the arc.

The model incorporates temperature-dependent thermal properties, including thermal conductivity, specific heat capacity, and density, to more accurately simulate the nonlinear thermal behavior of the material under rapid heating and cooling cycles. The mesh model, as shown in Figure 8b, adopts a refined mesh in the clad region and at the substrate–clad interface to ensure accurate resolution of steep thermal gradients and detailed molten pool geometry. Coarser mesh elements are used in regions distant from the heat source to reduce computational cost without compromising simulation accuracy.

Heat loss due to convection and radiation is considered at the top surface exposed to ambient air, while other boundaries are assumed adiabatic. The transient solution of the heat transfer equation allows tracking of temperature evolution and molten pool morphology as the heat source traverses along the cladding path. The resulting temperature fields enable detailed observation and quantification of key features such as molten pool depth, width, and shape, which are critical indicators of cladding quality.

This thermal FEM model, enhanced by the physically representative Goldak double-ellipsoid heat source, provides a robust foundation for investigating the influence of process parameters—such as arc current, scanning speed, and heat input—on the size and morphology of the molten pool in PTA cladding. Through accurate meshing, realistic boundary conditions, and advanced heat source modeling, the model supports systematic optimization of the PTA process by linking operating parameters to molten pool behavior.

### 4.2. Evolution of Molten Pool Morphology During Single-Track PTA Cladding

Plasma Transferred Arc (PTA) cladding is characterized by rapid thermal cycles, resulting in steep temperature gradients within the molten pool. These gradients lead to spatial variations in surface tension, which act as the primary driving forces for fluid flow within the pool. Such convective behavior significantly influences the thermal field distribution, the surface morphology of the deposited layer, and the metallurgical bonding quality at the clad–substrate interface. Therefore, in-depth investigation of the temperature field evolution during the cladding process is crucial for optimizing cladding quality and process parameters.

The thermal cycle associated with PTA cladding involves rapid heating and cooling, particularly at the trailing edge of the molten pool. As the pool solidifies, steep thermal gradients and high cooling rates (~10^4^–10^5^ K/s) promote the formation of hard intermetallic compounds. Specifically, primary borides and carbides—such as CrB, Cr_7_C_3_, or Cr_23_C_6_—precipitate early during solidification, followed by the development of a Ni-rich γ matrix during the final stages. These microstructural features are known to enhance hardness and wear resistance. Although detailed phase transformation modeling is beyond the current scope, the observed cooling kinetics suggest a solidification sequence consistent with prior metallurgical studies on Ni–Cr–B–Si based alloys. This interpretation aligns with the observed bead morphology and mechanical performance.

**Figure 9:**
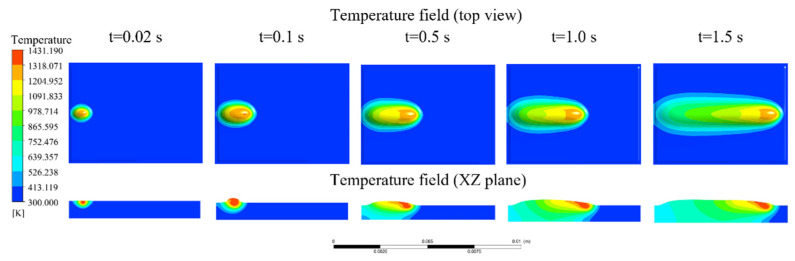
Evolution of the temperature field in the molten pool at different time intervals. (High-resolution top-view and cross-sectional temperature field distributions of the molten pool at various time points during plasma transferred arc (PTA) cladding).

A single-track simulation was performed under the following process parameters: arc current of 130 A, powder feed rate of 25 g/min, traverse speed of 88 mm/min, torch oscillation speed of 2600 mm/min, and oscillation width of 7.5 mm. Figure 9 presents top-view snapshots of the temperature field and corresponding XZ cross-sectional temperature distributions at different time intervals. At *t* = 0.02 s, the plasma arc initiates interaction with the substrate surface. Owing to the short exposure duration, heat absorption is limited, and the peak temperature reaches only ~1153 K—below the liquidus temperature of both the alloy powder (~1523 K) and substrate (~1653 K). Consequently, no molten pool is formed at this stage. By *t* = 0.1 s, the peak temperature increases to approximately 1756 K, surpassing the melting points of both materials. At this point, partial melting of the fed powder occurs, forming a nascent bulged region on the substrate. Although a molten pool is established, the overall temperature remains relatively low and spatial growth is limited.

As cladding progresses, the cumulative heat input leads to a rise in the central pool temperature due to thermal conduction. At *t* = 0.5 s, the molten pool reaches a quasi-steady state, characterized by a distinct comet-like morphology. Beyond this point, the central temperature exhibits minimal fluctuation. This stabilization is attributed to the elevated substrate temperature, which decreases the material’s thermal conductivity. Consequently, convective heat transfer within the molten pool and radiative heat loss from the surface become the dominant heat dissipation mechanisms. Once the heat input balances with convective and radiative losses, the thermal and geometric state of the molten pool stabilizes.

Cross-sectional analysis of the temperature field reveals that the leading edge of the molten pool is consistently lower in height than the solidified trailing region. This is attributed to a pronounced temperature gradient between the front and rear regions of the molten pool, which induces internal thermal convection. The convective flow transports molten material from the leading edge backward, thereby reducing the local build height at the front.

**Figure 10:**
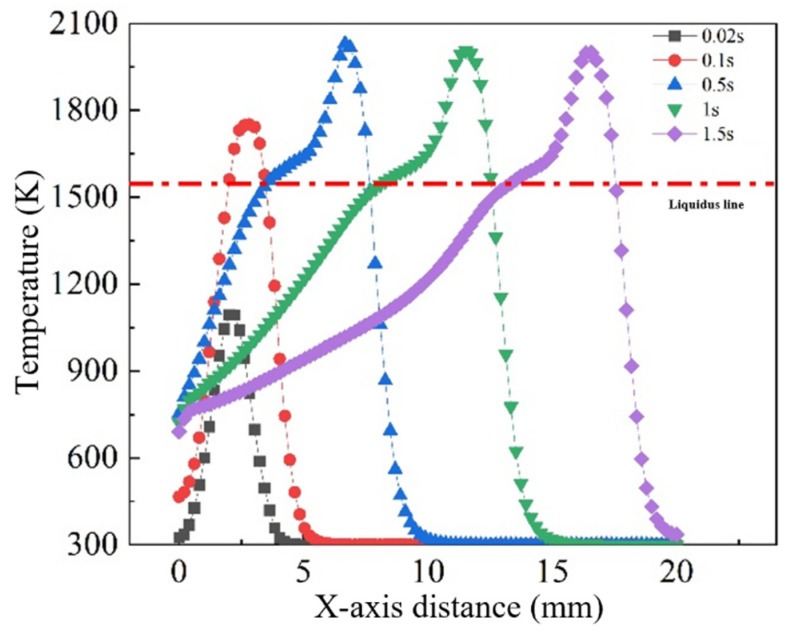
Temperature distribution along the X-axis at different times.

To quantitatively assess thermal evolution along the scanning path, temperature profiles along the X axis at various time steps were extracted (Figure 10). Each profile displays a pronounced peak, with the trailing side exhibiting a gentler slope, while the leading side features a steeper gradient. This asymmetry arises because the rear region, having already passed under the heat source, experiences gradual heat dissipation, while the leading edge—still in contact with the cooler substrate—exhibits a steeper thermal gradient due to intense energy transfer and localized absorption.

Although the maximum pool temperature theoretically aligns with the center of the heat source, the simulation results indicate a lag in the peak temperature relative to the actual arc center. This discrepancy is attributed to thermal inertia: the front edge of the arc interacts with the substrate for a shorter duration, resulting in inadequate heating, while the middle and rear regions accumulate more thermal energy. Furthermore, fluid convection within the molten pool redistributes heat rearward, further contributing to the offset.

**Figure 11:**
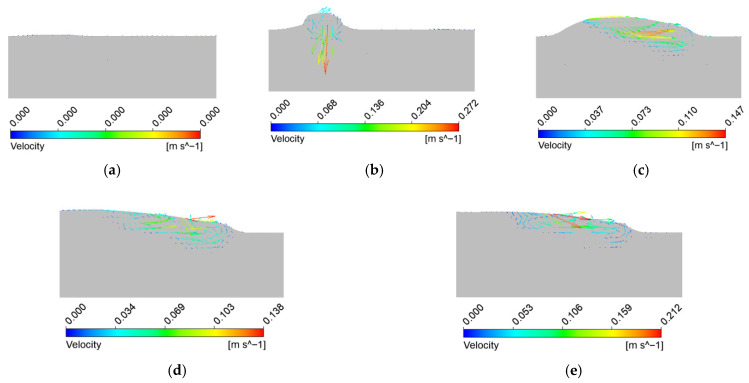
Cloud diagrams of the XZ cross-section of the molten pool flow field at different times. (**a**) *t* = 0.02 s; (**b**) *t* = 0.1 s; (**c**) *t* = 0.5 s; (**d**) *t* = 1 s; (**e**) *t* = 1.5 s.

Figure 11 illustrates the evolution of the flow field in the XZ cross-section at different cladding times. The arrows represent flow direction, and the color contours denote flow velocity magnitudes. At *t* = 0.02 s, no molten pool exists, hence no flow is observed. By *t* = 0.1 s, thermal convection emerges due to Marangoni effects. Molten metal in the high-temperature central zone flows outward toward both sides, forming symmetric clockwise and counterclockwise flow loops. At this stage, the maximum flow velocity reaches approximately 0.272 m/s.

As the process continues, the molten pool expands and advances along the scanning direction, forming a trailing tail (Figure 11b). Convection intensifies, but the central pool zone shows relatively lower velocities due to the more uniform temperature distribution. In contrast, the highest flow velocities are observed near the solid–liquid boundaries, particularly at the interface with the substrate. This observation is consistent with the findings of Zhao Mingjuan et al. and suggests that the steep temperature gradients and solidification dynamics near the periphery intensify convective motion.

The relatively low viscosity of molten metal in the high-temperature pool center results in smoother, more uniform flow, while abrupt changes in viscosity near the pool edges, driven by sharp thermal gradients, increase local velocities. Beyond *t* = 1 s, the maximum flow velocity stabilizes around 0.19 m/s, in line with the thermal stabilization observed earlier. However, due to persistent surface tension gradients, the peak velocities remain concentrated near the surface and edges of the molten pool.

### 4.3. Experimental Validation of the Thermo-Mechanical Numerical Model

To evaluate the accuracy of the developed thermo-mechanical numerical model for plasma transferred arc (PTA) cladding, a series of validation experiments were carried out. A single-track cladding sample was produced and subsequently analyzed using both macroscopic imaging and non-destructive testing techniques. The resulting geometric features of the cladding layer were then compared with the numerical simulation outputs.

**Figure 12:**
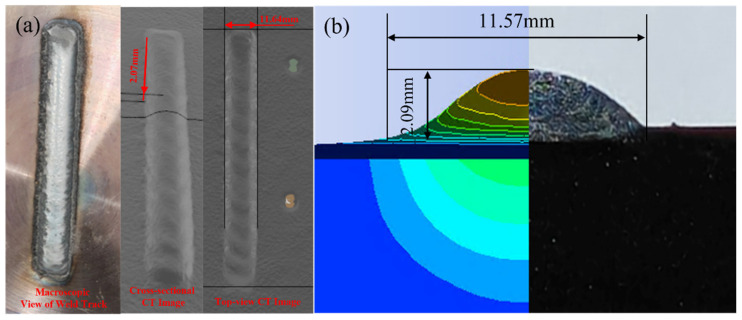
Morphological characterization of a single-track plasma cladding sample. (**a**) Macroscopic and X-ray CT images for width and thickness measurement; (**b**) Comparison of numerical simulation contour and metallographic cross-section to verify simulation accuracy.

As shown in Figure 12, the cladding track was first visually inspected (Figure 12a, left) to assess the surface morphology and overall weld bead quality. The internal structure and geometric characteristics were then captured using X-ray computed tomography (CT). The scanning process was conducted using a Nikon XT H 225 ST system, (Nikon Metrology, Tring, UK) a high-resolution industrial CT scanner suitable for metal additive processes. The raw data were reconstructed and analyzed using VGStudio MAX 3.4 software (Volume Graphics GmbH, Germany), which enabled the precise extraction of the cladding width and height from the CT slices.

The experimental measurements revealed a cladding width of 11.64 mm and a height (or buildup thickness) of 2.07 mm, as indicated in the CT image. In comparison, the numerical simulation predicted a width of 11.57 mm and a height of 2.09 mm, as shown in Figure 12b, which overlays the simulation contour on the metallographic cross-section.

To quantitatively assess the model’s accuracy, relative errors were calculated as follows:

$$\mathrm{Cladding}\;\mathrm{width}\;\mathrm{error}:\;\frac{\left|11.64-\right.\left.11.57\right|}{11.64}\times 100\%=0.06\%$$

$$\mathrm{Cladding}\;\mathrm{height}\;\mathrm{error}:\;\frac{\left|2.07-\right.\left.2.09\right|}{2.07}\times 100\%=0.97\%$$

As shown in Table 5, the comparison between the simulation and experimental results reveals excellent agreement in both cladding width and height. The absolute error in width is 0.07 mm, corresponding to a relative error of 0.60%, while the height shows an absolute error of 0.02 mm with a relative error of 0.97%. The computed tomography (CT) scanner used has a resolution of approximately 50 μm, corresponding to a measurement uncertainty of roughly ±0.02 mm, which is small relative to the bead dimensions. These small deviations—both below 1%—indicate that the thermo-mechanical numerical model is highly accurate in predicting the geometrical outcomes of the PTA cladding process.

Both key dimensions showed relative errors below 1%, indicating excellent agreement between the simulation results and experimental measurements. These minimal discrepancies validate that the model accurately captures the essential physical phenomena involved in the PTA cladding process, such as heat conduction, molten pool dynamics, and solidification behavior.

Moreover, the good overlap between the simulated contour lines and the experimental metallographic profile further confirms the model’s reliability for predicting cladding geometry. Therefore, the developed thermo-mechanical model can be considered a robust tool for process optimization and parametric sensitivity analysis in PTA cladding applications.

## 5. Influencing Factors and Evaluation of Coating Morphology in Plasma Cladding

### 5.1. Influence of Process Parameters on Bead Geometry in Plasma Transferred Arc (PTA) Cladding

**Figure 13:**
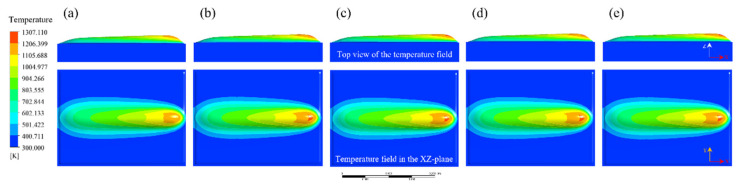
Evolution of the temperature field during Plasma Transferred Arc (PTA) cladding at 100 A under different process parameters: (**a**) 15 g/min powder feed rate, 66 mm/min welding speed, 1800 mm/min torch oscillation speed, and 4.0 mm oscillation width; (**b**) 20 g/min, 77 mm/min, 2600 mm/min, 10.0 mm; (**c**) 25 g/min, 88 mm/min, 3400 mm/min, 6.0 mm; (**d**) 30 g/min, 99 mm/min, 2200 mm/min, 12.0 mm; (**e**) 35 g/min, 110 mm/min, 3000 mm/min, 8.0 mm.

**Figure 14:**
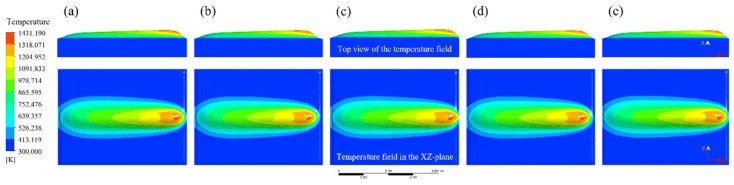
Evolution of the temperature field during Plasma Transferred Arc (PTA) cladding at 115 A under different process parameters: (**a**) 15 g/min powder feed rate, 77 mm/min welding speed, 2200 mm/min torch oscillation speed, and 6.0 mm oscillation width; (**b**) 20 g/min, 88 mm/min, 3000 mm/min, 12.0 mm; (**c**) 25 g/min, 99 mm/min, 1800 mm/min, 8.0 mm; (**d**) 30 g/min, 110 mm/min, 2600 mm/min, 4.0 mm; (**e**) 35 g/min, 66 mm/min, 3400 mm/min, 10.0 mm.

**Figure 15:**
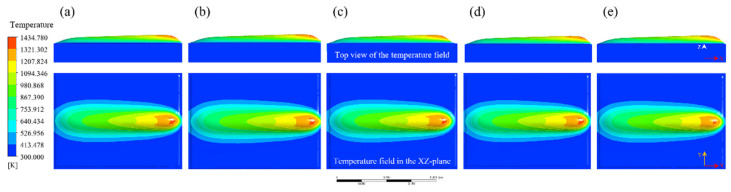
Evolution of the temperature field during Plasma Transferred Arc (PTA) cladding at 130 A under different process parameters: (**a**) 15 g/min powder feed rate, 88 mm/min welding speed, 2600 mm/min torch oscillation speed, and 8.0 mm oscillation width; (**b**) 20 g/min, 99 mm/min, 3400 mm/min, 4.0 mm; (**c**) 25 g/min, 110 mm/min, 2200 mm/min, 10.0 mm; (**d**) 30 g/min, 66 mm/min, 3000 mm/min, 6.0 mm; (**e**) 35 g/min, 77 mm/min, 1800 mm/min, 12.0 mm.

**Figure 16:**
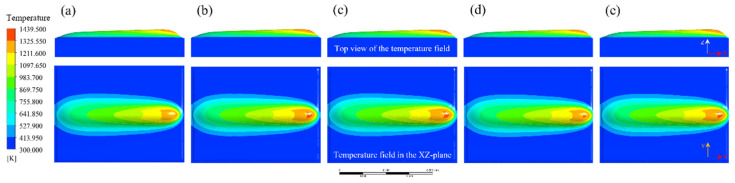
Evolution of the temperature field during Plasma Transferred Arc (PTA) cladding at 145 A under different process parameters: (**a**) 15 g/min powder feed rate, 99 mm/min welding speed, 3000 mm/min torch oscillation speed, and 10.0 mm oscillation width; (**b**) 20 g/min, 110 mm/min, 1800 mm/min, 6.0 mm; (**c**) 25 g/min, 66 mm/min, 2600 mm/min, 12.0 mm; (**d**) 30 g/min, 77 mm/min, 3400 mm/min, 8.0 mm; (**e**) 35 g/min, 88 mm/min, 2200 mm/min, 4.0 mm.

**Figure 17:**
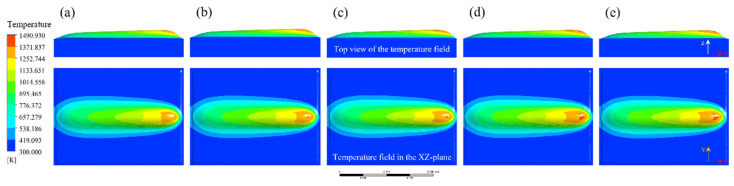
Evolution of the temperature field during Plasma Transferred Arc (PTA) cladding at 160 A under different process parameters: (**a**) 15 g/min powder feed rate, 110 mm/min welding speed, 3400 mm/min torch oscillation speed, and 12.0 mm oscillation width; (**b**) 20 g/min, 66 mm/min, 2200 mm/min, 8.0 mm; (**c**) 25 g/min, 77 mm/min, 3000 mm/min, 4.0 mm; (**d**) 30 g/min, 88 mm/min, 1800 mm/min, 10.0 mm; (**e**) 35 g/min, 99 mm/min, 2600 mm/min, 6.0 mm.

In order to investigate the influence of process parameters on bead geometry in Plasma Transferred Arc (PTA) cladding, a three-dimensional transient numerical simulation was conducted. The simulations were performed under varying current levels (100–160 A) with different combinations of powder feed rate, welding speed, and oscillation parameters. The spatial distribution of temperature fields was extracted to characterize the thermal behavior of the molten pool, as illustrated in Figure 13, Figure 14, Figure 15, Figure 16 and Figure 17. From the simulated temperature fields, key geometric attributes of the clad beads, namely bead height (H), bead width (W), and penetration depth (P), were derived. To quantitatively assess the geometric quality, a morphology index was defined as M = (H·W)/P, where a higher value corresponds to improved buildup and wider coverage with reduced dilution. This combined analysis of thermal field evolution and morphology index enables a systematic evaluation of the parameter–geometry relationship in PTA cladding.

At 100 A, the simulated temperature field, as shown in Figure 13, indicates that the molten pool attains moderate peak temperatures of approximately 1300 K. Under conditions of lower powder feed rate and welding speed, the pool exhibits a relatively narrow and deep profile, with thermal concentration near the trailing region. With increasing powder feed rate and oscillation width, the isothermal contours expand laterally, and the molten pool gradually transitions into a wider and shallower morphology.

The corresponding bead geometry, as listed in Table 6, confirms these observations: bead heights remain within the range of 1.87–2.16 mm, while bead widths are in the range of 10.67–11.84 mm. In contrast, penetration depth decreases markedly from 1.34 mm to 0.50 mm, reflecting a redistribution of heat input from the vertical to the transverse direction. As a result, the morphology index increases substantially from 14.89 to 49.55, indicating improved bead geometry with enhanced buildup, broader coverage, and reduced dilution.

At 115 A, the simulated temperature field, as shown in Figure 14, reveals a notable increase in peak temperature to approximately 1430 K. Compared with 100 A, the isothermal contours display a broader lateral distribution, reflecting enhanced heat spreading across the molten pool. This lateral expansion promotes wider surface coverage while maintaining relatively moderate penetration.

The corresponding bead geometry, as listed in Table 7, demonstrates that bead heights increase slightly within the range of 2.00–2.48 mm, and bead widths remain close to 10–12 mm, whereas penetration depths vary between 0.75 and 1.22 mm. Consequently, the morphology index falls within the range of 18.09–35.38, suggesting that at this current level the geometric quality achieves a favorable balance between buildup and penetration. These results indicate that a moderate increase in current improves wetting and coverage without causing excessive dilution, thereby producing bead morphologies with desirable formability and stability.

At 130 A, the simulated temperature field, as shown in Figure 15, indicates that the molten pool reaches maximum temperatures exceeding 1430 K and exhibits a pronounced lateral expansion compared with lower current levels. The pool morphology becomes wider and more flattened, suggesting stronger heat input and redistribution in the transverse direction.

The corresponding bead geometry, as listed in Table 8, shows that bead widths remain relatively stable within 10.38–12.0 mm, while bead heights increase to as high as 2.60 mm. In contrast, penetration depths fluctuate more strongly in the range of 0.76–1.19 mm, reflecting enhanced sensitivity of the molten pool to process variations. As a result, the morphology index varies between 19.84 and 33.89, indicating that although increased current promotes bead buildup and lateral spreading, the risk of excessive penetration becomes more pronounced, thereby requiring precise control of oscillation parameters to maintain desirable bead geometry.

At 145 A, the simulated temperature field, as shown in Figure 16, demonstrates further intensification of the thermal field, with more flattened isothermal contours and a molten pool that becomes increasingly laterally extended. This is accompanied by increased penetration depths, ranging from 0.98 to 1.20 mm, indicating that the additional heat input drives the molten pool deeper into the substrate.

The corresponding bead geometry, as listed in Table 9, shows that bead heights and widths remain comparable to those observed at 130 A, with values of approximately 2.0–2.57 mm and 10.11–12.0 mm, respectively. However, the morphology index decreases to 19.05–27.77, reflecting that the advantages of stable buildup and lateral coverage are offset by excessive penetration. These results suggest that at higher current levels, excessive heat input compromises geometric quality by promoting dilution, even when bead height and width remain within favorable ranges.

At 160 A, the simulated temperature field, as shown in Figure 17, reveals that the molten pool attains maximum temperatures of approximately 1490 K and exhibits the greatest lateral spread among all investigated current levels. While this broadening enhances surface coverage, it is accompanied by a marked increase in penetration depth, reaching values up to 1.49 mm.

The corresponding bead geometry, as listed in Table 10, indicates that bead heights and widths remain relatively stable in the ranges of 1.97–2.60 mm and 11.77–12.0 mm, respectively. However, the morphology index decreases to 20.68–28.13, which is notably lower than that achieved at 100–130 A. These results suggest that although increased current promotes wider pool morphology and stable buildup, the excessive penetration induced at 160 A compromises overall geometric quality and increases the risk of dilution.

Taken together, the results clearly demonstrate the progressive influence of current on molten pool behavior and bead morphology during PTA cladding. At lower current levels (100–115 A), the molten pool exhibits moderate peak temperatures and a favorable wide–shallow morphology, resulting in high morphology indices due to shallow penetration combined with stable bead buildup. At intermediate current levels (around 130 A), lateral spreading is further enhanced and bead height increases; however, greater fluctuations in penetration depth make the morphology index more sensitive to parameter variations. At higher current levels (145–160 A), the intensified thermal field promotes deeper penetration, which reduces the morphology index, even though bead width and height remain relatively stable. Overall, these findings indicate that optimal geometric quality is achieved at low to intermediate current levels (100–130 A), where wide coverage and shallow penetration are maintained. In contrast, higher current levels (145–160 A) increase the risk of excessive dilution, unless carefully controlled through oscillation dynamics and other process parameters.

### 5.2. Analysis of the Morphology Index Response to Key Process Parameters

To systematically evaluate how process parameters shape bead geometry, we defined a quantitative Morphology Index (M) and mapped it across the design space using heatmaps and three-dimensional (3D) surface plots derived from the experimental dataset.

**Figure 18:**
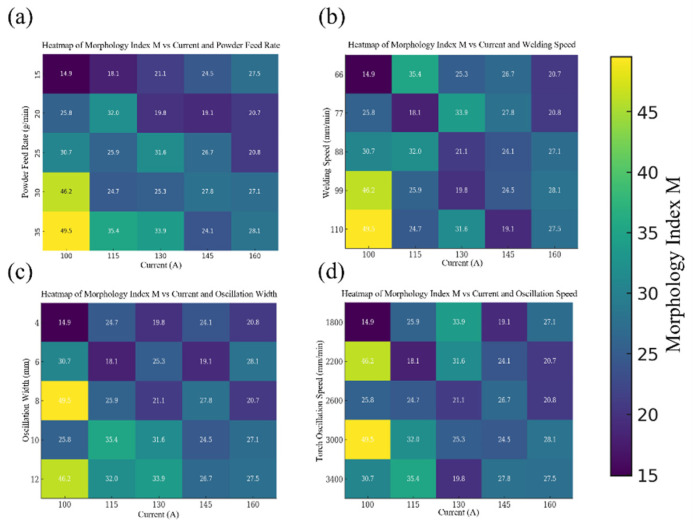
Heatmaps of the Morphology Index (M) in plasma cladding, illustrating the effect of current in combination with: (**a**) powder feed rate, (**b**) welding speed, (**c**) oscillation width, and (**d**) oscillation speed. (These high-resolution heatmaps show the interaction between current and other process parameters, where warmer colors (indicating higher M values) represent improved bead morphology—characterized by a smoother, more uniform surface).

Figure 18 summarizes the single-factor pairings with current. Across all panels, larger M values correspond to smoother surface morphology and more uniform bead geometry. A consistent pattern is that the lowest current level (100 A) yields substantially higher M values, especially when combined with high powder feed rate (35 g/min), low welding speed (66 mm/min), wide oscillation width (8–12 mm), or moderate oscillation speed (≈2200 mm/min). For example, in subplots (a) and (d), the highest M values (49.5 and 46.2) occur at 100 A, indicating that reduced current enhances deposition stability when material supply or oscillation control is sufficient. Beyond ~130 A, M generally decreases or plateaus, suggesting that excessive heat input degrades morphological quality via deeper penetration and less controlled solidification. These trends indicate an optimal process window in which current is balanced against auxiliary parameters to maximize clad quality within the tested ranges.

**Figure 19:**
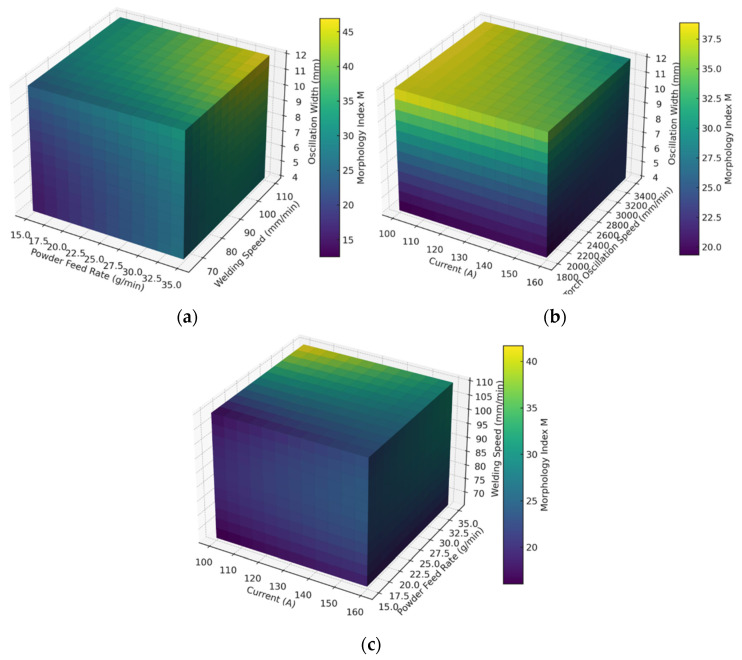
Three-dimensional plots of the Morphology Index (M) under different process-parameter combinations in plasma cladding. (**a**) Powder Feed Rate–Welding Speed–Oscillation Width; (**b**) Current–Torch Oscillation Speed–Oscillation Width; (**c**) Current–Powder Feed Rate–Welding Speed.

Building on this, Figure 19 highlights multi-parameter couplings. In Figure 19a (Powder Feed Rate–Welding Speed–Oscillation Width), M increases with higher welding speed and wider oscillation width, particularly when the powder feed rate is moderate-to-high (30–35 g/min), implying that adequate material supply and distributed heat input mitigate dilution and improve bead stability. In Figure 19b (Current–Torch Oscillation Speed–Oscillation Width), current and oscillation dynamics emerge as critical: low-to-moderate current (100–115 A) combined with moderate oscillation speed (≈2400–2800 mm/min) and wide oscillation width (8–12 mm) produces the highest M, reflecting the benefits of controlled heat input and effective oscillatory redistribution. Conversely, at higher currents (>145 A), M declines, consistent with over-melting and loss of geometric control. In Figure 19c (Current–Powder Feed Rate–Welding Speed), the interplay of current, feed rate, and travel speed confirms that low-to-moderate current (100–130 A), high welding speed (100–110 mm/min), and relatively high powder feed rate (30–35 g/min) provide the most favorable balance, ensuring sufficient deposition without overheating.

From a mechanistic standpoint, these results can be rationalized by thermo-fluid behavior in the molten pool. At low-to-moderate current, the heat input sustains a stable pool without inducing excessive penetration. Under these conditions, Marangoni convection—driven by surface-tension gradients—promotes lateral redistribution of molten metal, enhancing pool uniformity and reducing the risk of undercutting or keyhole formation. A wider oscillation width with moderate oscillation speed further improves heat and mass distribution, suppresses local overheating, and stabilizes the solidification front. In contrast, higher current increases penetration depth and destabilizes the pool, leading to uneven solidification and lower M. Likewise, insufficient powder feed (relative to heat input) or excessively low welding speed (prolonged thermal exposure) promotes geometric instability.

Overall, within the explored parameter space, M is maximized by low-to-moderate current (100–115 A), high welding speed (100–110 mm/min), wide oscillation width (8–12 mm), and high powder feed rate (30–35 g/min). A representative optimum is 100 A current, 105 mm/min welding speed, 35 g/min powder feed rate, 10 mm oscillation width, and 2600 mm/min oscillation speed, which together ensure superior geometric stability, minimized dilution, and improved clad quality. This combination balances deposition rate and heat input while stabilizing molten-pool convection and solidification, yielding optimal bead morphology with enhanced surface smoothness. These findings provide practical guidance for process optimization and theoretical insight into the coupling between thermal input and oscillation dynamics in plasma cladding.

## 6. Conclusions

In this study, the key knowledge gaps identified in the Introduction have been addressed: we have developed an integrated morphology index to evaluate bead geometry, and combined orthogonal experimental design with numerical simulation to optimize single-pass PTA cladding. The results confirm that arc current is the dominant factor and identify an optimal parameter set (low-to-moderate current, high speed, high feed, wide oscillation) that minimizes dilution. These findings satisfy the original objectives and provide practical guidelines for PTA process control.

Results indicate that the arc current is the dominant factor controlling molten pool stability. Low-to-moderate currents (100–115 A) favor wide–shallow pools and high morphology indices, while higher currents (145–160 A) increase penetration and dilution, degrading quality. At intermediate levels (130 A), bead buildup improves but penetration fluctuations become significant.

Multi-factor analysis shows that optimal morphology is achieved under low-to-moderate current, high welding speed (100–110 mm/min), relatively high powder feed rate (30–35 g/min), wide oscillation width (8–12 mm), and moderate oscillation speed (~2600 mm/min). A representative optimal set (100 A, 105 mm/min, 35 g/min, 10 mm, 2600 mm/min) ensures balanced heat–material input, stabilized molten pool convection, and minimized dilution.

Overall, the integration of experiments, simulations, and morphological evaluation provides practical guidelines for process optimization and offers theoretical insights into thermo-fluid interactions in PTA cladding. The established framework supports the development of adaptive control strategies to further improve clad quality and reliability in surface engineering applications. Due to resource constraints, each condition was replicated once; prior experience suggests the cladding bead dimensions vary by less than ~5% for identical runs. The computed tomography (CT) scanner has a resolution of ~50 μm, corresponding to an uncertainty of roughly ±0.02 mm in measured geometry, which is small relative to the bead dimensions. Future work will include additional repeats to quantify variability.

## Figures and Tables

**Table 1:** Chemical composition of Colmonoy 56 powder (wt.%).

Element	B	C	Cr	Fe	Ni	Si	Others
Content (wt.%)	1.9	0.90	18.0	5.4	Bal.	5.3	trace

**Table 2:** Chemical composition of 45Cr9Si3 electroslag remelted steel (wt.%).

Element	C	Si	Mn	P	S	Ni	Cr	Cu	Fe
Content (wt.%)	0.43	3.14	0.50	0.023	0.002	0.21	8.62	0.033	Bal.

**Table 3:** PTA cladding process parameters.

Parameter	Value
Main arc current (A)	120
Traverse speed (mm·min^−1^)	120
Powder feed rate (g·min^−1^)	8.0
Oscillation amplitude (mm)	5
Plasma gas flow (L·min^−1^)	3 (Ar/2%H_2_)
Shielding gas flow (L·min^−1^)	10
Preheat temperature (°C)	150
Ambient temperature (K)	293.15

**Table 4:** Orthogonal design matrix and corresponding process parameters for PTA cladding experiments.

Number	Current (A)	Powder Feed Rate (g/min)	Welding Speed (mm/min)	Torch Oscillation Speed (mm/min)	Oscillation Width (mm)
1	100.0	15.0	66.0	1800.0	4.0
2	100.0	20.0	77.0	2600.0	10.0
3	100.0	25.0	88.0	3400.0	6.0
4	100.0	30.0	99.0	2200.0	12.0
5	100.0	35.0	110.0	3000.0	8.0
6	115.0	15.0	77.0	2200.0	6.0
7	115.0	20.0	88.0	3000.0	12.0
8	115.0	25.0	99.0	1800.0	8.0
9	115.0	30.0	110.0	2600.0	4.0
10	115.0	35.0	66.0	3400.0	10.0
11	130.0	15.0	88.0	2600.0	8.0
12	130.0	20.0	99.0	3400.0	4.0
13	130.0	25.0	110.0	2200.0	10.0
14	130.0	30.0	66.0	3000.0	6.0
15	130.0	35.0	77.0	1800.0	12.0
16	145.0	15.0	99.0	3000.0	10.0
17	145.0	20.0	110.0	1800.0	6.0
18	145.0	25.0	66.0	2600.0	12.0
19	145.0	30.0	77.0	3400.0	8.0
20	145.0	35.0	88.0	2200.0	4.0
21	160.0	15.0	110.0	3400.0	12.0
22	160.0	20.0	66.0	2200.0	8.0
23	160.0	25.0	77.0	3000.0	4.0
24	160.0	30.0	88.0	1800.0	10.0
25	160.0	35.0	99.0	2600.0	6.0

**Table 5:** Comparison between simulation and experimental results with error analysis.

Metric	Experimental	Simulation	Absolute Error	Relative Error
Cladding Width (mm)	11.64	11.57	0.07	0.60%
Cladding Height (mm)	2.07	2.09	0.02	0.97%

**Table 6:** Measured bead geometry and calculated morphology index (M) in PTA cladding.

Number	Bead Height H (mm)	Bead Width W (mm)	Penetration Depth P (mm)	Morphology Index M
1	1.87	10.67	1.34	14.89
2	1.94	11.59	0.87	25.84
3	2.11	11.64	0.8	30.7
4	2.07	11.84	0.53	46.24
5	2.16	11.47	0.5	49.55

**Table 7:** Measured bead geometry and calculated morphology index (M) in PTA cladding.

Number	Bead Height H (mm)	Bead Width W (mm)	Penetration Depth P (mm)	Morphology Index M
6	2.06	10.71	1.22	18.09
7	2.0	12.0	0.75	31.99
8	2.09	10.91	0.88	25.92
9	2.03	9.85	0.81	24.67
10	2.48	12.0	0.84	35.38

**Table 8:** Measured bead geometry and calculated morphology index (M) in PTA cladding.

Number	Bead Height H (mm)	Bead Width W (mm)	Penetration Depth P (mm)	Morphology Index M
11	2.03	11.44	1.1	21.12
12	1.97	10.38	1.03	19.84
13	2.06	11.64	0.76	31.56
14	2.51	12.0	1.19	25.28
15	2.6	12.0	0.92	33.89

**Table 9:** Measured bead geometry and calculated morphology index (M) in PTA cladding.

Number	Bead Height H (mm)	Bead Width W (mm)	Penetration Depth P (mm)	Morphology Index M
16	2.0	12.0	0.98	24.48
17	2.09	10.11	1.11	19.05
18	2.54	12.0	1.14	26.71
19	2.48	12.0	1.07	27.77
20	2.57	11.24	1.2	24.06

**Table 10:** Measured bead geometry and calculated morphology index (M) in PTA cladding.

Number	Bead Height H (mm)	Bead Width W (mm)	Penetration Depth P (mm)	Morphology Index M
21	1.97	12.0	0.86	27.48
22	2.57	12.0	1.49	20.68
23	2.51	11.77	1.42	20.79
24	2.6	12.0	1.15	27.11
25	2.54	11.97	1.08	28.13

## Data Availability

The original contributions presented in this study are included in the article. Further inquiries can be directed to the corresponding author.
